# West Nile Virus Seroprevalence in the Italian Tuscany Region from 2016 to 2019

**DOI:** 10.3390/pathogens10070844

**Published:** 2021-07-05

**Authors:** Serena Marchi, Emanuele Montomoli, Simonetta Viviani, Simone Giannecchini, Maria A. Stincarelli, Gianvito Lanave, Michele Camero, Caterina Alessio, Rosa Coluccio, Claudia Maria Trombetta

**Affiliations:** 1 Department of Molecular and Developmental Medicine, University of Siena, 53100 Siena, Italy; emanuele.montomoli@unisi.it (E.M.); simonetta.viviani@unisi.it (S.V.); caterina.alessio@student.unisi.it (C.A.); rosa.coluccio@gmail.com (R.C.); trombetta@unisi.it (C.M.T.); 2 VisMederi S.r.l., 53100 Siena, Italy; 3 Department of Experimental and Clinical Medicine, University of Florence, 50134 Firenze, Italy; simone.giannecchini@unifi.it (S.G.); mariastincarelli@gmail.com (M.A.S.); 4 Department of Veterinary Medicine, University of Bari, 70010 Valenzano, Italy; gianvito.lanave@gmail.com (G.L.); michele.camero@uniba.it (M.C.)

**Keywords:** West Nile virus, antibody, seroprevalence, Italy

## Abstract

Although in humans West Nile virus is mainly the cause of mild or sub-clinical infections, in some cases a neuroinvasive disease may occur predominantly in the elderly. In Italy, several cases of West Nile virus infection are reported every year. Tuscany was the first Italian region where the virus was identified; however, to date only two cases of infection have been reported in humans. This study aimed at evaluating the prevalence of antibodies against West Nile virus in the area of Siena Province to estimate the recent circulation of the virus. Human serum samples collected in Siena between 2016 and 2019 were tested for the presence of antibodies against West Nile virus by ELISA. ELISA positive samples were further evaluated using immunofluorescence, micro neutralization, and plaque reduction neutralization assays. In total, 1.9% (95% CI 1.2–3.1) and 1.4% (95% CI 0.8–2.4) of samples collected in 2016–2017 were positive by ELISA and immunofluorescence assay, respectively. Neutralizing antibodies were found in 0.7% (95% CI 0.3–1.5) of samples. Additionally, 0.9% (95% CI 0.4–1.7) and 0.65% (95% CI 0.3–1.45) of samples collected in 2018–2019 were positive by ELISA and immunofluorescence assay, respectively. The prevalence of neutralizing antibodies was 0.5% (95% CI 0.2–1.3). Although no human cases of West Nile infection were reported in the area between 2016 and 2019 and virus prevalence in the area of Siena Province was as low as less than 1%, the active asymptomatic circulation confirms the potential concern of this emergent virus for human health.

## 1. Introduction

West Nile virus (WNV) is an emerging virus involving birds and Culex mosquitoes in its transmission cycle. Spillover events from this cycle involve mammalian hosts, in particular horses and humans, considered dead-end hosts [1]. In humans, although most infections (about 80%) are asymptomatic, those with a clinical manifestation present with a mild febrile illness, known as West Nile fever, are often underdiagnosed. In some cases, especially among the elderly, a more severe infection may develop as West Nile neuroinvasive disease (WNND), associated with significant morbidity and mortality [2]. As WNND is observed in less than 1% of infected subjects, the frequency of subclinical infections leads to an underestimation of the actual circulation of the virus.

In Italy, WNV is endemic in the Northern regions, where several cases of infection in humans are reported every year. However, in recent years the virus has expanded its distribution with cases also registered in Central and Southern Italy [3].

WNV was reported for the first time in Italy in 1998 among horses residing in wetland areas of Tuscany, Central Italy [4], while the first human case of WNND was detected ten years later, in 2008, in the Emilia-Romagna region, Northern Italy [5]. However, a retrospective study showed that in 2007 a woman living in Tuscany was infected with WNV [6], demonstrating that the virus was already circulating among humans in Central Italy before its isolation in Emilia-Romagna causing unrecognized human disease.

Following the identification of the first human cases of WNV infection, specific WNND surveillance systems were set up in the Emilia-Romagna and Veneto regions [7], followed by the implementation of national veterinary and human surveillance plans [8]. Surveillance activities include entomological, veterinary and human surveillance to be carried out from June to November, identified as the high-risk transmission period. Since 2008, WNV circulation has been reported in 10 Italian regions (Emilia-Romagna, Veneto, Lombardy, Sardinia, Sicily, Friuli Venezia Giulia, Piedmont, Tuscany, Basilicata, Apulia). From 2008 to 2017 a total of 231 cases of human WNND were reported [9].

**Figure 1:**
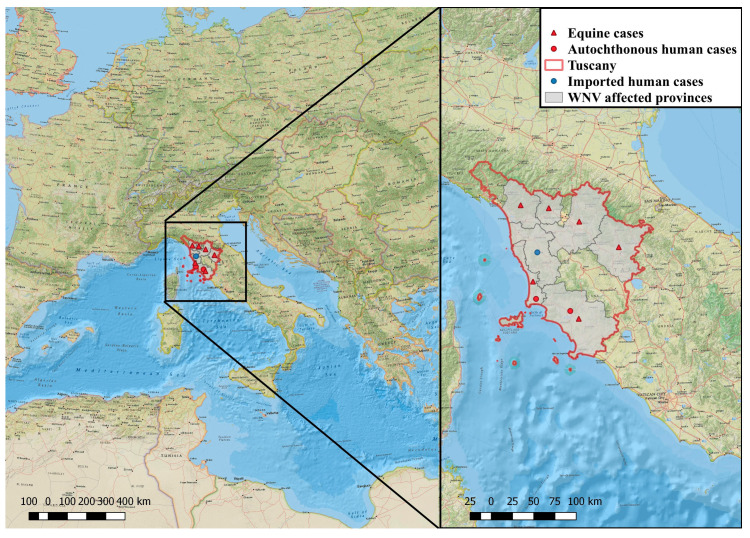
West Nile disease equine and autochthonous and imported human reported cases retrieved in Tuscany since 1998. The territories affected with West Nile virus (WNV) are colored in grey and those that are WNV-free are colorless. The maps were generated with Esri ArcGis Desktop 10.6.1 (www.esri.com, accessed on 2 June 2021). Red triangles, equine cases; Red circles, autochthonous human cases; Blue circle, imported human cases.

The presence of WNV in Tuscany was reported in horses in 1998 [4], in 2009 [10], and in 2016 [11]. Although Tuscany was the first Italian region where the presence of WNV human infections was identified already in 2007 [6], since then only one imported case in 2011 in the province of Pisa [12] and two WNND cases in 2017 in the province of Livorno, a coastal area of the region, were reported [13] (Figure 1).

In Italy, human cases of WNV infection are usually detected starting from July and peaking in August–September. However, in 2018 the transmission season started earlier with the first detection of WNV from a pool of Culex mosquitoes in the Veneto region on the 7th of June [14], and the first confirmed human case was reported just 9 days later in the same province [15]. As of December 2018, a total of 577 confirmed cases of human infection were reported in the Veneto, Emilia-Romagna, Lombardy, Piedmont, Sardinia, and Friuli Venezia Giulia regions. In that very year, veterinary surveillance reported an increase in the circulation of WNV in mosquitoes, birds and horses in nine Italian regions (Emilia-Romagna, Veneto, Lombardy, Sardinia, Friuli Venezia Giulia, Piedmont, Lazio, Basilicata, and Apulia) [16].

Since 2018, the coastal provinces of Tuscany have been considered endemic areas [17], and since 2019, the province of Siena has been included among areas at high risk of transmission by national surveillance plans [18,19]. However, to date no cases of infection have been reported by routine surveillance activities.

Reports of epidemics in equine holdings suggest the circulation of WNV in the Tuscany region. However, human cases reported by routine surveillance are few. To date, limited data are available on the prevalence of WNV in the region.

The primary aim of this study was to evaluate the prevalence of WNV antibodies in the Siena Province area, in the Tuscany region, to estimate the recent circulation of WNV in an area where no infection cases have ever been reported by surveillance activities. The second aim was to investigate any variation in WNV prevalence in the province after the increase in transmission observed during the 2018 season.

## 2. Results

A total of 1800 samples, 879 for the years 2016–2017 and 921 for the years 2018–2019, were tested. The median age of all subjects whose serum samples were included in the study was 51 years, 50 years (age range 20–93 years) and 51 years (age range 20–94 years) for those sampled in 2016–2017 and in 2018–2019, respectively.

Collected samples were stratified by year of collection (2016–2017 and 2018–2019) and further stratified by sex and age group (20–60 and >60 years old). IgG borderline and positive samples identified by ELISA, immunofluorescence assay (IFA) and micro-neutralization (MN)/plaque reduction neutralization (PRN) assays in different years of collection by age group are reported in Table 1.

Out of the 879 samples collected in 2016–2017, 17 (1.9%, 95% confidence interval (CI) 1.2–3.1) samples were ELISA IgG positive or borderline. Comparisons of ELISA IgG positive results with sex (*p* = 0.83) and age groups (*p* = 0.69) did not show any statistical significance. Twelve samples (1.4%, 95% CI 0.8–2.4) were confirmed IgG positive by IFA. IFA IgG positive results were also not statistically associated with sex (*p* = 0.85) and age groups (*p* = 0.71). Six samples were positive by MN and PRN assays, thus showing a total prevalence of 0.7% (95% CI 0.3–1.5) of samples with neutralizing antibodies. MN/PRN positive results lack any statistical significance with sex (*p* = 0.87) and age groups (*p* = 0.74).

Out of the 879 samples collected in 2016–2017, 92 were also tested by ELISA IgM. Four samples were found positive (4.35%, 95% CI 1.36–11.0), one of which was also positive for ELISA IgG, IFA IgG, and neutralizing antibodies.

Out of the 921 samples collected in 2018–2019, eight (0.9%, 95% CI 0.4–1.7) samples were positive by ELISA IgG. Comparisons of ELISA IgG positive results with sex (*p* = 0.18) and age groups (*p* = 0.71) did not yield any statistical significance. Six samples (0.65%, 95% CI 0.3–1.45) were confirmed IgG positive by IFA. IFA IgG positive results were also not statistically associated with sex (*p* = 0.23) and age group (*p* = 0.77). Five samples were positive by MN and PRN for a total prevalence of 0.5% (95% CI 0.2–1.3). MN/PRN positive results lack any statistical significance by comparison with sex (*p* = 0.26) and age groups (*p* = 0.79).

Table 2 shows a summary of results with the characteristics of subjects who showed neutralizing antibodies to WNV. The median age was 57 years (age range 30–91 years).

In the univariate logistic regression model, the independent variables, sex and age group, did not show, consistently, statistically significant associations with IgG positive and borderline results for both 2016–2017 and 2018–2019 years of collection. In the multivariate logistic regression model, the independent variables confirmed the lack of association with IgG positive or borderline results (Table 3).

## 3. Discussion

To our knowledge, this is the first study on the prevalence of WNV antibodies in the Tuscany region. The results show that, although between 2016 and 2019 WNV prevalence in the area of Siena Province was as low as less than 1%, in 2016 and 2017 WNV was actively circulating, as shown by the finding of specific IgM suggestive of recent infection. Moreover, in some of the positive subjects the presence of antibodies with neutralizing may suggest a potentially protective immunity.

To date, the presence of WNV in Tuscany has been reported in horses in 1998 [4], in 2009 [10], and in 2016 [11], while WNV infection in humans was retrospectively diagnosed in 2007 [6] and two cases of WNND were reported in 2017 [13].

This is the first study showing that cases of human infection by WNV have occurred in the Siena area and may be considered of some relevance as no human cases have been reported by routine surveillance in the study period 2016–2019. In addition, it appears that WNV circulation was not an occasional finding but was detected in both 2016–2017 and 2018–2019 periods studied. The reason why no cases of WNV infection were reported in the study period from the Siena area may be due to the fact that WNV is often the cause of mild or sub-clinical infection, which may lead to misdiagnosis and underreporting of the disease.

Other serological studies conducted on the general population or blood donors in other areas of Italy where the circulation of WNV has been registered (Lombardy, Emilia-Romagna, and Veneto regions) [20,21,22,23,24] have found similar prevalence to this study. The same median age of 57 years we found in our study was observed in WNV positive blood donors in the Veneto region [25], probably due to the fact that our study most likely includes asymptomatic WNV infections or mild symptomatic infections.

Similar WNV prevalence studies performed in other European countries and in the Mediterranean Basin detected neutralizing antibodies in 1.5% and 2.34% of the population in Greece [26] and in Hungary [27], respectively, while the results of this study are more in line with the prevalence observed in Bulgaria [28].

Historically, surveillance activities detected WNV infection cases in the Northern East areas of Italy. However, over the years, increasing numbers of West Nile fever cases have been reported from other areas, suggesting viral circulation expanding in the general population in areas previously considered naïve [3], as shown in our study. In fact, the population included in this study, although not selected for the purpose, better represents the general population than that included in other epidemiological studies performed in blood donors or in international travelers [21,22,24,29,30].

This study has some limitation. Samples were collected for purposes different from the aim of this study; thus, no information on clinical findings such as fever or neurological signs and symptoms was available. Our study population did not include subjects younger than 20 years of age.

Recently, among mosquito-borne flaviviruses, with birds as reservoir hosts, circulating in different areas of Europe, Usutu virus has been reported to possess serological cross-reactions with WNV [31]. In our study, WNV-positive samples were not tested for Usutu virus neutralizing antibodies; therefore, a possible cross-reactivity between the two viruses cannot be totally excluded. Usutu virus circulation in the Tuscany region has been reported only in mosquito pools in 2018 and 2019 from two provinces in the Northern area of the region, and no animal or human cases were reported from routine surveillance activities [16,32]. Moreover, IgM ELISA antibodies have been detected in some samples of this study. IgM ELISA is usually considered to be more specific than IgG, with a lower cross-reactivity with other flaviviruses [33,34]. Taking into account the data on the circulation of both viruses in the area and the results obtained from all the serological assays performed in this study, the specific reaction to WNV can be reasonably assumed.

This study shows for the first time the active circulation in humans of WNV that occurred between 2016 and 2019 in the Siena area, an area considered not at high risk until 2019. Although the prevalence of WNV is limited as compared to other neurotropic arboviruses, such as Toscana virus [35], it appears to have acquired an established transmission pattern between 2016 and 2019.

In conclusion, WNV infection appears to be more widespread in the area of Siena than has been detected so far, and it is possible that some cases of infection are underdiagnosed and underreported. Taking into consideration the trend of the expansion of WNV in Central Italy, the absence of reported WNV human cases in the Siena area should not limit the application of preventive measures and epidemiological surveillance, as the low prevalence of antibodies does not prevent outbreaks of WNV disease in the future.

## 4. Materials and Methods

### 4.1. Study Population

The study was performed with samples available at the sera bank of the Molecular Epidemiology Laboratory of the University of Siena, Italy. Human serum samples are residual samples collected from a local laboratory in the province of Siena between 2016 and 2019. Samples were anonymously collected and stored in compliance with Italian ethics law. For each serum sample, information only on age, sex, place and year of sampling was available.

A total of 1800 samples were randomly selected from the sera bank: 879 for the years 2016–2017 and 921 for the years 2018–2019.

### 4.2. ELISA and Immunofluorescence Assay

All samples were tested for the presence of IgG antibodies against WNV by use of “West Nile Virus IgG” (DIA.PRO, Milano, Italy) commercial ELISA kit. Testing was performed according to manufacturer’s instructions, and test results were calculated by means of a cut-off value determined with the following formula: Cut-off = optical density (OD) of the negative control + 0.250. Samples were considered positive when the ratio between the OD of the sample and that of the cut-off was >1.1, and negative when the ratio between the OD of the sample and that of the cut-off was <0.9. Samples with a ratio between 0.9 and 1.1 were considered borderline.

Out of 879 samples collected in 2016–2017, 92 were also tested for the presence of IgM antibodies by use of “West Nile Virus IgM” (DIA.PRO, Milano, Italy) commercial ELISA kit. IgM ELISA testing was performed on all the ELISA IgG positive and borderline samples and on a subset of ELISA IgG negative samples. Testing was performed according to manufacturer’s instructions and results were calculated as for ELISA IgG kit.

ELISA borderline and positive samples were further tested by “Anti-West Nile virus (IgG)” (EUROIMMUN, Lübeck, Germany) IFA commercial kit, following manufacturer’s instructions. Samples were tested with 2-fold dilutions from 1:50 to 1:6400. The IFA titer was defined as the highest serum dilution showing fluorescence, as reported by manufacturer’s instructions.

All IgG and IgM ELISA and IgG IFA positive samples were further tested by MN and PRN assays.

### 4.3. Micro Neutralization and Plaque Reduction Neutralization Assays

The cell substrate used was Vero E6 (African green monkey kidney cell line; ATCC^®^ CRL-1586™) propagated in Dulbecco’s Modified Eagle’s Medium (DMEM; Sigma-Aldrich, St. Louis, MO, USA) supplemented with 10% Fetal Bovine Serum (FBS; Sigma-Aldrich, St. Louis, MO, USA). The WNV strain (lineage 2) viral stock, consisting of cell-free supernatants of acutely infected Vero E6 cells, was stored at −80 °C until use. Prior to the MN and PRN test, WNV was titrated for 50% tissue culture infectious dose (TCID_50_) and plaque forming unit (PFU) using Vero E6 cells, and all serum samples were heat-inactivated at 56 °C for 30 min.

MN assay was performed by exposing (1:1) serial twofold dilutions of heat-inactivated serum in DMEM (1:10 to 1:320) to 100 TCID_50_ of WNV. After 1-h incubation at 37 °C in 5% CO_2_ atmosphere, 50 µL of the serum/virus mixture was plated on each well of a 96-well plate covered by Vero E6 cell monolayers (10^4^ cells/well), and incubated for 1 h at 37 °C, 5% CO_2_. Then, 50 µL of DMEM was added on each well and the plate was incubated for 4 days up to the appearance of an easy detectable cytopathic effect in control cultures (cell monolayers exposed to WNV). Additionally, IgG serum negative to WNV was used as control. The antibody titer was defined as the reciprocal of the highest dilution of the test serum sample, which showed at least 50% neutralization.

PRN assay was performed on heat-inactivated serum samples by exposing (1:1) serial twofold dilutions of them in DMEM (1:10 to 1:320) to 100 PFU of WNV. After incubation for 1 h at 37 °C, 5% CO_2_ atmosphere, 300 µL of the serum/virus mixture was plated on each well of 6-well plates seeded with 2.5 × 10^5^ Vero E6 cells and incubated 1 h at 37 °C. Then, the overlay medium composed of 0.5% Sea Plaque Agarose (Lonza, Basel, Switzerland) diluted in propagation medium was added to each well. After 4 days of incubation at 37 °C, the monolayers were fixed with methanol (Carlo Erba Chemicals, Milan, Italy) and stained with 0.1% crystal violet (Carlo Erba Chemicals, Milan, Italy) and the viral titers were calculated by PFU counting. Percent of PRN was calculated by dividing the average PFU of viral serum treated samples by the average of viral positive control. All experiments were repeated at least twice. All experimental procedures were conducted under biosafety level 3 containment.

### 4.4. Statistical Analysis

Categorical dichotomous data (sex and age group) and discrete data (IgG ELISA, IFA, MN/PRN assays results) were defined as categorical dichotomous data, described as counts and percentages and evaluated by Chi-square test. The relations between the IgG positivity of each assay as a dependent categorical dichotomous variable defined as a dummy variable and independent factors (sex and age group) were evaluated by logistic regression model, and OR, 95% CI, and *p*-values were assessed. In the univariate logistic regression model, all the factors related to IgG positivity were investigated as independent variables. The statistically significant independent variables were assessed in the multivariate logistic regression model using Wald test and stepwise method for the selection of *p*-value. Statistical significance was set at *p* < 0.05.

Data from statistical analyses were performed with the software GraphPad Prism v.6.0.0 (GraphPad Software, San Diego, CA, USA).

### 4.5. Geographic Methods

The spatial distribution of WND human and equine reported cases was mapped using QGIS 3.6.0 [36]. The shapefile of Tuscany region (WGS84 UTM32N) was retrieved from the National Institute of Statistics (ISTAT) [37]. The national geographic map was used as basemap to relate the study area to the European region.

## Figures and Tables

**Table 1:** ELISA IgG, IFA IgG, and MN/PRN borderline and positive samples by years of collection and age group. Data on sex and age group of collected and positive samples were summarized as counts and percentages and 95% confidence intervals were calculated.

Study Population	2016–2017						2018–2019					
	M	F	Total	ELISA IgG	IFA IgG	MN/PRN	M	F	Total	ELISA IgG	IFA IgG	MN/PRN
20–60 years	255 (43.8)	327 (56.2)	582 (66.2)	10 (1.7%, 0.9–3.2)	8 (1.4%, 0.65–2.7)	3 (0.5%, 0.1–1.6)	307 (50.1)	306 (49.9)	613 (66.6)	6 (1.0%, 0.4–2.2)	4 (0.65%, 0.2–1.7)	3 (0.5%, 0.1–1.5)
>60 years	161 (54.2)	136 (45.8)	297 (33.8)	7 (2.4%, 1.05–4.9)	4 (1.35%, 0.4–3.5)	3 (1.0%, 0.2–3.1)	154 (50.0)	154 (50.0)	308 (33.4)	2 (0.65%, 0.0–2.5)	2 (0.65%, 0.0–2.5)	2 (0.65%, 0.0–2.5)
Total	416 (47.3)	463 (52.7)	879	17 (1.9%, 1.2–3.1)	12 (1.4%, 0.8–2.4)	6 (0.7%, 0.3–1.5)	461 (50.1)	460 (49.9)	921	8 (0.9%, 0.4–1.7)	6 (0.65%, 0.3–1.45)	5 (0.5%, 0.2–1.3)

95% CI, 95% confidence interval; IFA, immunofluorescence assay; MN, micro neutralization; PRN, plaque reduction neutralization.

**Table 2:** Information of subjects (years of collection, age and sex) and serologic results (ELISA, IFA, MN and PRN titer) of the samples showing WNV neutralizing antibodies by MN/PRN assays.

Years	Subject	Age Group	Age (Years)	Sex	ELISA IgG	IFA IgG	MN Titer	PRN Titer
2016–2017	1	>60	61	F	Positive	1/100	20	10
	2	>60	64	M	Positive	1/6400	20	40
	3	20–60	57	F	Borderline	1/50	20	10
	4	>60	91	F	Borderline	1/50	20	20
	5	20–60	42	F	Positive	1/50	10	10
	6	20–60	33	F	Positive	1/800	10	40
2018–2019	7	20–60	52	M	Positive	1/400	10	40
	8	>60	75	M	Positive	1/50	10	20
	9	20–60	30	M	Positive	1/3200	20	10
	10	20–60	32	M	Positive	1/6400	40	40
	11	>60	85	F	Positive	1/100	20	20

IFA, immunofluorescence assay; MN, micro neutralization; PRN, plaque reduction neutralization.

**Table 3:** Results for the univariate logistic regression model and the multivariate logistic regression model by independent variables (sex and age group).

Years of Collection	Independent Variable	Univariate Logistic Regression Model			Multivariate Logistic Regression Model		
		OR	95% CI	*p* Value	OR	95% CI	*p* Value
2016–2017	Sex	0.80	0.30–2.08	0.64	0.82	0.31–2.16	0.69
	Age group	1.39	0.52–3.68	0.51	1.36	0.51–3.62	0.54
2018–2019	Sex	0.28	0.06–1.37	0.12	0.28	0.06–1.37	0.12
	Age group	0.57	0.12–2.74	0.48	0.57	0.12–2.74	0.48

OR, odd ratio; 95% CI, 95% confidence interval.

## References

[1] Beck C., Jimenez-Clavero M.A., Leblond A., Durand B., Nowotny N., Leparc-Goffart I., Zientara S., Jourdain E., Lecollinet S. (2013). Flaviviruses in Europe: Complex Circulation Patterns and Their Consequences for the Diagnosis and Control of West Nile Disease. Int. J. Environ. Res. Public Health.

[2] Gould E., Solomon T. (2008). Pathogenic flaviviruses. Lancet.

[3] Rizzo C., Napoli C., Venturi G., Pupella S., Lombardini L., Calistri P., Monaco F., Cagarelli R., Angelini P., Bellini R. (2016). West Nile virus transmission: Results from the integrated surveillance system in Italy, 2008 to 2015. Eurosurveillance.

[4] Autorino G.L., Battisti A., Deubel V., Ferrari G., Forletta R., Giovannini A., Lelli R., Murri S., Scicluna M.T. (2002). West Nile virus Epidemic in Horses, Tuscany Region, Italy. Emerg. Infect. Dis..

[5] Rossini G., Cavrini F., Pierro A., Macini P., Finarelli A.C., Po C., Peroni G., Di Caro A., Capobianchi M.R., Nicoletti L. (2008). First human case of West Nile virus neuroinvasive infection in Italy, September 2008—Case report. Eurosurveillance.

[6] Cusi M.G., Roggi A., Terrosi C., Savellini G.G., Toti M. (2011). Retrospective Diagnosis of West Nile Virus Infection in a Patient with Meningoencephalitis in Tuscany, Italy. Vector Borne Zoonotic Dis..

[7] Rizzo C., Vescio F., Declich S., Finarelli A.C., Macini P., Mattivi A., Rossini G., Piovesan C., Barzon L., Palù G. (2009). West Nile virus transmission with human cases in Italy, August–September 2009. Eurosurveillance.

[8] Ministero della Salute Sorveglianza della Malattia di West Nile in Italia—2010. https://www.trovanorme.salute.gov.it/norme/renderNormsanPdf?anno=0&codLeg=34923&parte=1%20&serie=.

[9] Moirano G., Richiardi L., Calzolari M., Merletti F., Maule M. (2019). Recent rapid changes in the spatio-temporal distribution of West Nile Neuro-invasive Disease in Italy. Zoonoses Public Health.

[10] Monaco F., Savini G., Calistri P., Polci A., Pinoni C., Bruno R., Lelli R. (2011). 2009 West Nile disease epidemic in Italy: First evidence of overwintering in Western Europe?. Res. Vet. Sci..

[11] Scaramozzino P., Carvelli A., Bruni G., Cappiello G., Censi F., Magliano A., Manna G., Ricci I., Rombolà P., Romiti F. (2021). West Nile and Usutu viruses co-circulation in central Italy: Outcomes of the 2018 integrated surveillance. Parasites Vectors.

[12] Rizzo C., Salcuni P., Nicoletti L., Ciufolini M.G., Russo F., Masala R., Frongia O., Finarelli A.C., Gramegna M., Gallo L. (2012). Epidemiological surveillance of West Nile neuroinvasive diseases in Italy, 2008 to 2011. Eurosurveillance.

[13] Istituto Superiore di Sanità Sorveglianza Integrata del West Nile e Usutu Virus, Bollettino N. 13 del 9 Novembre 2017. https://www.epicentro.iss.it/westnile/bollettino/Bollettino%20WND_08.11.2017.pdf.

[14] Istituto Superiore di Sanità Sorveglianza integrata del West Nile e Usutu Virus, Bollettino N. 1 del 28 Giugno 2018. https://www.epicentro.iss.it/westnile/bollettino/Bollettino%20WND_n.1%2028.06.2018.pdf.

[15] Riccardo F., Monaco F., Bella A., Savini G., Russo F., Cagarelli R., Dottori M., Rizzo C., Venturi G., Di Luca M. (2018). An early start of West Nile virus seasonal transmission: The added value of One Heath surveillance in detecting early circulation and triggering timely response in Italy, June to July 2018. Eurosurveillance.

[16] Istituto Superiore di Sanità Sorveglianza Integrata del West Nile e Usutu virus, Bollettino N. 18 del 15 Novembre 2018. https://www.epicentro.iss.it/westnile/bollettino/Bollettino%20WND_%20N.%2018%20%2015.%2011%202018.pdf.

[17] Ministero della Salute Piano Nazionale Integrato di Sorveglianza e Risposta ai Virus West Nile e Usutu—2018. https://www.trovanorme.salute.gov.it/norme/renderNormsanPdf?anno=2018&codLeg=65084&parte=1%20&serie=null.

[18] Ministero della Salute Piano Nazionale Integrato di Prevenzione, Sorveglianza e Risposta ai Virus West Nile e Usutu—2019. https://www.trovanorme.salute.gov.it/norme/renderNormsanPdf?anno=2019&codLeg=68806&parte=1%20&serie=null.

[19] Ministero della Salute Piano Nazionale di Prevenzione, Sorveglianza e Risposta alle Arbovirosi (PNA) 2020–2025. https://www.salute.gov.it/imgs/C_17_pubblicazioni_2947_allegato.pdf.

[20] Faggioni G., De Santis R., Pomponi A., Grottola A., Serpini G.F., Meacci M., Gennari W., Tagliazucchi S., Pecorari M., Monaco F. (2018). Prevalence of Usutu and West Nile virus antibodies in human sera, Modena, Italy, 2012. J. Med. Virol..

[21] Pierro A., Gaibani P., Manisera C., Dirani G., Rossini G., Cavrini F., Ghinelli F., Ghinelli P., Finarelli A.C., Mattivi A. (2011). Seroprevalence of West Nile Virus–Specific Antibodies in a Cohort of Blood Donors in Northeastern Italy. Vector Borne Zoonotic Dis..

[22] Pierro A., Gaibani P., Spadafora C., Ruggeri D., Randi V., Parenti S., Finarelli A.C., Rossini G., Landini M.P., Sambri V. (2013). Detection of specific antibodies against West Nile and Usutu viruses in healthy blood donors in northern Italy, 2010–2011. Clin. Microbiol. Infect..

[23] Gaibani P., Pierro A., Lunghi G., Farina C., Toschi V., Matinato C., Orlandi A., Zoccoli A., Almini D., Landini M.P. (2013). Seroprevalence of West Nile virus antibodies in blood donors living in the metropolitan area of Milan, Italy, 2009–2011. New Microbiol..

[24] Pezzotti P., Piovesan C., Barzon L., Cusinato R., Cattai M., Pacenti M., Piazza A., Franchin E., Pagni S., Bressan S. (2011). Prevalence of IgM and IgG antibodies to West Nile virus among blood donors in an affected area of north-eastern Italy, summer 2009. Eurosurveillance.

[25] Barzon L., Pacenti M., Franchin E., Pagni S., Lavezzo E., Squarzon L., Martello T., Russo F., Nicoletti L., Rezza G. (2013). Large Human Outbreak of West Nile Virus Infection in North-Eastern Italy in 2012. Viruses.

[26] Hadjichristodoulou C., Pournaras S., Mavrouli M., Marka A., Tserkezou P., Baka A., Billinis C., Katsioulis A., Psaroulaki A., Papa A. (2015). West Nile Virus Seroprevalence in the Greek Population in 2013: A Nationwide Cross-Sectional Survey. PLoS ONE.

[27] Nagy A., Szöllősi T., Takács M., Magyar N., Barabás É. (2019). West Nile Virus Seroprevalence Among Blood Donors in Hungary. Vector Borne Zoonotic Dis..

[28] Christova I., Panayotova E., Tchakarova S., Taseva E., Trifonova I., Gladnishka T. (2017). A nationwide seroprevalence screening for West Nile virus and Tick-borne encephalitis virus in the population of Bulgaria. J. Med. Virol..

[29] Loconsole D., Metallo A., De Robertis A.L., Morea A., Quarto M., Chironna M. (2018). Seroprevalence of Dengue Virus, West Nile Virus, Chikungunya Virus, and Zika Virus in International Travelers Attending a Travel and Migration Center in 2015–2017, Southern Italy. Vector Borne Zoonotic Dis..

[30] Barzon L., Pacenti M., Cusinato R., Cattai M., Franchin E., Pagni S., Martello T., Bressan S., Squarzon L., Cattelan A.M. (2011). Human cases of West Nile Virus Infection in north-eastern Italy, 15 June to 15 November 2010. Eurosurveillance.

[31] Llorente F., García-Irazábal A., Pérez-Ramírez E., Cano-Gómez C., Sarasa M., Vázquez A., Jiménez-Clavero M.Á. (2019). Influence of flavivirus co-circulation in serological diagnostics and surveillance: A model of study using West Nile, Usutu and Bagaza viruses. Transbound. Emerg. Dis..

[32] Istituto Superiore di Sanità Sorveglianza Integrata del West Nile e Usutu Virus, Bollettino N. 16 del 25 Novembre 2019. https://www.epicentro.iss.it/westnile/bollettino/Bollettino-WND-N16-25nov2019.pdf.

[33] Tardei G., Ruta S., Chitu V., Rossi C., Tsai T.F., Cernescu C. (2000). Evaluation of immunoglobulin M (IgM) and IgG enzyme immunoassays in serologic diagnosis of West Nile Virus infection. J. Clin. Microbiol..

[34] Martin D.A., Biggerstaff B.J., Allen B., Johnson A.J., Lanciotti R.S., Roehrig J.T. (2002). Use of immunoglobulin m cross-reactions in differential diagnosis of human flaviviral encephalitis infections in the United States. Clin. Diagn. Lab. Immunol..

[35] Marchi S., Trombetta C.M., Kistner O., Montomoli E. (2017). Seroprevalence study of Toscana virus and viruses belonging to the Sandfly fever Naples antigenic complex in central and southern Italy. J. Infect. Public Health.

[36] QGIS Development Team (2017). Quantum GIS Geographic Information System. Open Source Geospatial Foundation Project. http://qgis.osgeo.org..

[37] ISTAT Confini delle Unità Amministrative a Fini Statistici al 1° Gennaio 2021. https://www.istat.it/it/archivio/222527.

